# Mapping artisanal and small-scale mines at large scale from space with deep learning

**DOI:** 10.1371/journal.pone.0267963

**Published:** 2022-09-22

**Authors:** Mathieu Couttenier, Sebastien Di Rollo, Louise Inguere, Mathis Mohand, Lukas Schmidt

**Affiliations:** 1 GATE & Department of Economics, Ecole Normale Supérieure de Lyon, Lyon, France; 2 CEPR, Washington, DC, United States of America; University of Craiova, ROMANIA

## Abstract

Artisanal and small-scale mines (asm) are on the rise. They represent a crucial source of wealth for numerous communities but are rarely monitored or regulated. The main reason being the unavailability of reliable information on the precise location of the asm which are mostly operated informally or illegally. We address this issue by developing a strategy to map the asm locations using a convolutional neural network for image segmentation, aiming to detect surface mining with satellite data. Our novel dataset is the first comprehensive measure of asm activity over a vast area: we cover 1.75 million km^2^ across 13 countries in Sub-Tropical West Africa. The detected asm activities range from 0.1 ha to around 2, 000 ha and present a great diversity, yet we succeed in hitting acceptable compromises of performance, as achieving 70% precision while maintaining simultaneously 42% recall. Ultimately, the remarkable robustness of our procedure makes us confident that our method can be applied to other parts of Africa or the world, thus facilitating research and policy opportunities in this sector.

## Introduction

The number of people working in artisanal and small-scale mines (henceforth asm) has sharply increased in recent years, with around 44 million people worldwide now employed in the sector and an additional 150 million being indirectly dependent on it [1]. Almost one-third of these individuals live in Africa [2]. This number is conservative—estimated 70% of small-scale miners work informally—and is six times greater than the amount of people working in industrial mines. Moreover, about 20% of the global gold and diamond supply, 25% of tantalum and tin, and up to 80% of sapphire come from asm [3, 4].

In many anecdotal reports and case studies, asm are associated with workers without formal recognition or rights, health risks, child labour, environmental hazards (water and air pollution, deforestation), smuggling, illicit trade, insecurity and extortion by armed groups. On the other hand, the wealth of many communities heavily depends on asm activity, particularly in regions where economic alternatives are scarce and income from traditional livelihood activities (agriculture) are uncertain. Surprisingly, despite rich anecdotal evidence, case studies and field work investigating asm, the literature in social sciences is scarce when it comes to large and exhaustive studies. The lack of systematic evidence is concerning given the important policy relevance of the role asm activity plays in shaping economic development. Notably, the United Nations Development Program noted that “the mining industry has an unprecedented opportunity to mobilize significant human, physical, technological and financial resources to advance the SDGs (Sustainable Development Goals)” [5]. Still, the dearth of academic work and absence of empirical regularities is mainly due to the fact that many asm are operated informally or illegally, resulting in very limited information on this sector at the local level.

In this paper, we develop a suitable strategy to map locations and estimate the size of the asm activities by combining state-of-the-art machine learning techniques and remote sensing data. More specifically, we use a convolutional neural network (cnn) for pixel-level classification, a modified version of the deep learning model U-Net [6], which aims to detect surface mining using satellite data (Sentinel-2). One major challenge is handling the high-dimensional features of images and the scalability of a possible automatic solution. We train the U-Net model with millions of input data from high-resolution satellite images to output a probability for each pixel to be a mine. This gives us a precise estimation on whether a mining activity is taking place in a large array of landscapes and varieties of asm types. As proof of concept, we carefully evaluate this strategy through the lens of a region of 1.75 million km^2^ in the Sub-Tropical West Africa, partially covering 13 countries. Our final outcome represents the first comprehensive measure of the asm locations and sizes for a large region. Remarkably, the good performance of our model allows considering the generalization of our method to other regions in Africa.

Crucially, the data collection methodology covers a wide range of applications from i) policy makers and non-governmental organizations having a complete vision of all locations with asm activities, aiming to improve the monitoring of those locations in different dimensions such as health, environmental degradation, or taxation of the minerals extracted; to ii) research purposes. Our finding is also relevant for current debates over transparency in mineral supply chains.

## Methods

### Characteristics of the studied region and satellite images

**Fig 1:**
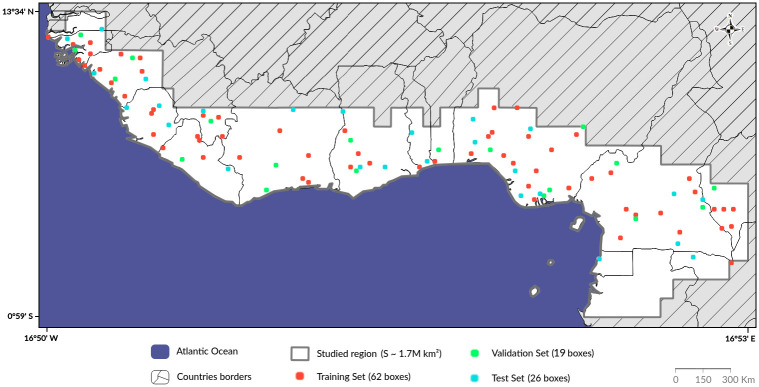
Studied region visualization. Mapping of the paper’s studied region (white) and presentation of the dispatched labeled dataset boxes, divided into *training set* (red), *validation set* (green) and *test set* (blue).

Our studied region covers a large area of 1.75 million km^2^ divided among 13 countries in the Sub-Tropical West Africa (Fig 1). This region encompasses three distinct biomes [7]: tropical and sub-tropical moist broad-leaf forests (41.1% of our sample region), a mixture of tropical and subtropical grasslands, savannas and shrub-lands (55.1%), and mangroves biome (3.8%) (S1 Fig). To minimize the cloud coverage density and to keep the vegetation colour relatively constant, we make use of satellite images from November to February. The spatial resolution of our images is 10*m* × 10*m* (Sentinel-2 satellite, European Space Agency Copernicus program). Sentinel-2 images combine 13 bands in the visible, near infrared, and short wave infrared part of the spectrum, ranging from 440 nm to 2200 nm, with different resolutions (S1 Table in S1 File). These bands perfectly fit our remote sensing application, as recent studies highlight the relevance of the near infrared bands to classify land cover mapping [8], along with the traditional visible bands (red, green and blue). For more information, see S1 Appendix in S1 File.

### Labelled dataset creation process

A crucial step of our methodology is the creation of a labelled dataset to train, tune and evaluate our model. For instance, during the training step, every time the model predicts a previously labelled region, it compares the ground truth label to the prediction made. The model parameters are then optimized by minimizing the error between these two. Even if qualitative initiatives exist to map mining activities [9], available data contains no information on location at pixel level, or the exact date of the labelling is missing or imprecise. The creation of our labelled dataset is particularly challenging as the identification of asm is related to class imbalance problem: while the number of asm has increased in the last years, they are still sparse and highly dispersed across space, likely representing less than 0.1% of the African continent.

Facing this challenge, we follow a sixth-steps strategy to create our reference dataset. First, we divide the region into a grid, each cell covering 14.5 km × 14.5 km (210 *km*^2^: 2.1 million pixels), with an overlap of 1 km on each side (S2 Fig). Second, to get a first idea about the potential asm distribution, we retrieve for each cell the density of “quarry” labels from the Open Street Map contributions. Indeed, pre-identifying boxes with a high density of “quarry” labels helps to overcome the challenge of the positive class’ sparseness. Third, we annotate a mixture of randomly chosen boxes with and without asm, looking for the right compromise between two conflicted purposes: i) including a subsequent number and variety of mining examples, while ii) maintaining our labelled dataset (relatively) representative of the class imbalance reality. After empirical exercises testing various asm proportions, and in coherency with our objective to first minimize the wrong predictions, we finally aimed to reach approximately 1% of asm in our dataset. However, it should not necessarily be the same value in other contexts (e.g. in other regions, or in fine-tuning an already pre-trained algorithm). Fourth, drawing lessons from our recurrent wrong prediction observations, we refine our dataset by manually select boxes containing particular features, such as rivers with sandy shores, railways and highways, and buildings under construction. Note that these three combined steps -2, 3 and 4- were not successive but iteratively operated together. In theory, they might be continually further-improved, by gradually enlarging and refining the current labelled dataset with a wide variety of landscapes, features and asm types. Fifth, for each selected box, given the fine-grained resolution of our images, we thoroughly label the area by imposing strict conditions for a pixel to be marked as an asm: i) limitation to a minimum size for a labelled mine (size > 1, 000 *m*^2^, i.e. minimum 10 pixels); ii) significant discordance in visible light ground colour compared to surroundings (e.g. to exclude the former asm that started re-vegetated); iii) obvious characteristics of mineral exploitation such as furrows/height holes, presence of pipelines and/or connection to roads; and iv) a typical signature in the temporal evolution of the feature’s shape. Sixth, in addition to these prerequisites, we use images with a resolution up to 60cm × 60cm from Google Earth to cross-validate that every asm location found on Sentinel-2 images is actually tangible asm. For a limited number of boxes—usually for very rural/remote regions -, the images’ resolution available in Google Earth was not sufficient and consequently, these boxes were excluded from the labelled dataset. Note that for each box, the labelling strategy was double-checked. In the end, our labelled dataset covers 23,337 km^2^ over 111 boxes (Fig 1).

### Dataset partition

To train and estimate the model performance, the labelled dataset is divided into three different partitions: i) the *training set* (55% / 13,035 *km*^2^) used to train the model; ii) the *validation set* (20% / 4,625 *km*^2^), with images the model has never seen during the training, which is used to tune the model parameters and to avoid over-fitting the training set. Also, the best weights are selected based on validation metrics only and iii) the *test set* (25% / 5,677 *km*^2^) with images the model has never seen before, to estimate the model’s performance. Given the limited amount of labelled data and the scarcity of asm areas in comparison to other landscapes, we select boxes for the *test set* to ensure that we have sufficient asm—in terms of variety of geological features and surface—to compute coherent metrics (see Results section), while keeping the maximum number of boxes in the *training set*. Boxes were also selected and split to fulfill several characteristics for each dataset: an equal distribution across regions, a consistent mine coverage proportion and to comprise various typical climate-regrouped landscapes: rivers, cities, agricultural fields, small mountains, coasts and wildlife.

### Pre-processing and model description

We start by implementing a pre-processing stage for Sentinel-2 images that includes atmospheric and geometrical corrections, normalization and data augmentation (see S2 and S3 Appendices in S1 File for further details). Following the recent advances in remote sensing, we make use of a modified version of the deep learning model U-Net [6], which is widely used in biomedical segmentation, semantic segmentation and remote-sensing applications [10–13]. Furthermore, a cnn like U-Net performs better than the traditional pixel-based methods [14] since the images’ contextual information is also considered, which is important as some mining activities can only be detected by the relevant understanding of their surroundings. Our model differs from the standard U-Net model by adding batch normalization layers [15] and dropout layers [16] to reduce the tendency toward over-fitting on the training data. In practice, over-fitting means that the model strictly memorizes examples seen during the training instead of learning from them, leading to a difficulty to generalize and usually a worse performance on new data. In S4 Appendix in S1 File, we provide more detailed information on the model and parameters used.

## Results

We investigate the performance of our model across several criteria by comparing the model’s predictions to our labelled *test set* composed of 5,677 km^2^ of images the model has never seen in training. We display a large array of statistics, from standard machine learning ones to metrics especially designed for our imbalanced dataset with respect to our project’s objectives.

### Performance metrics

**Fig 2:**
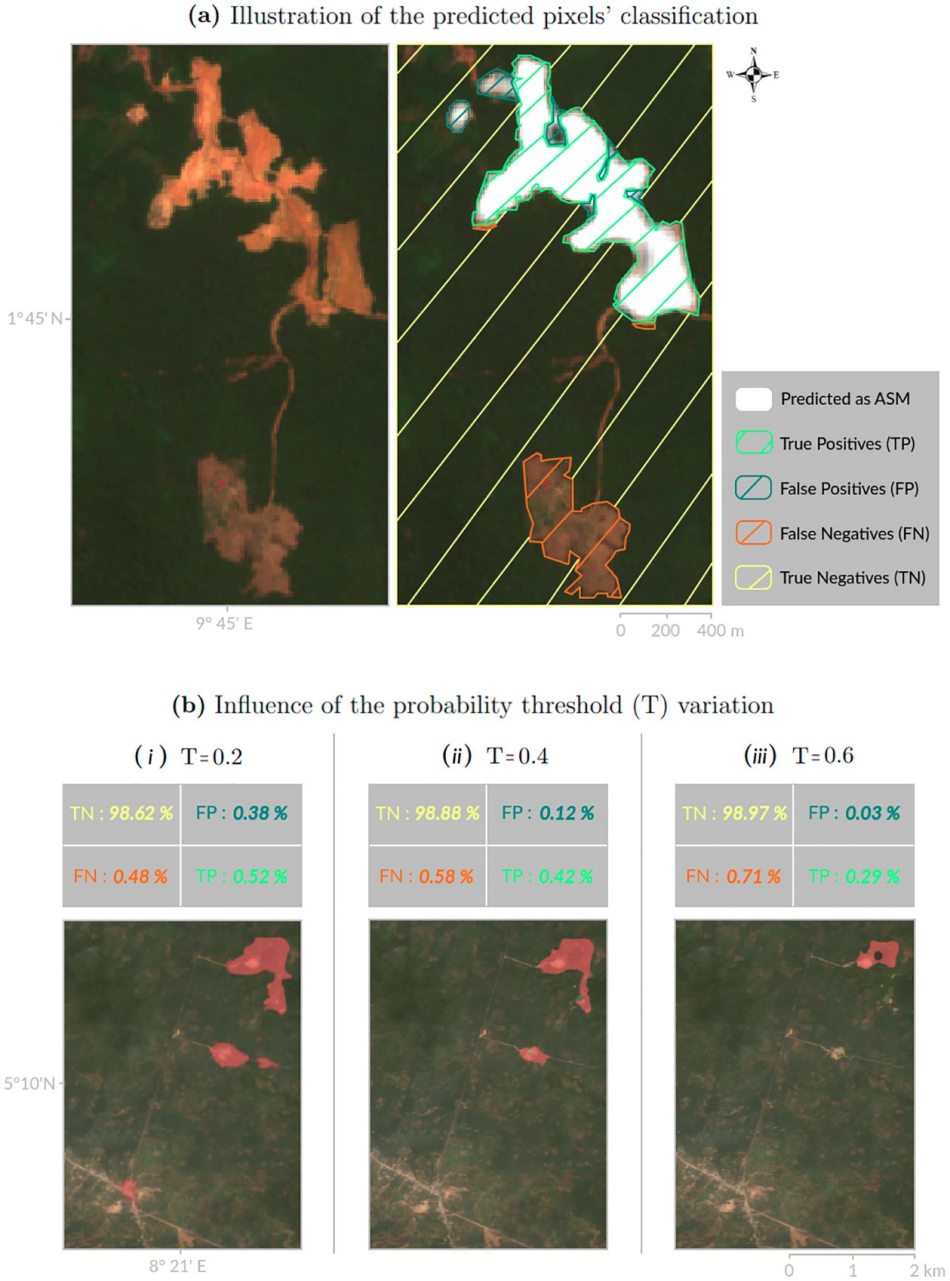
Classification of the predictions: Illustrations. (a) Illustration of the standard pixels classification derived from the comparison between prediction and reality (TP, FP, FN and TN, together forming the confusion matrix) from a sub-sample of our predicted *test set* in the region of Bata, Equatorial Guinea. (b) Confusion matrices computed for three distinct scenarios (defined in section Performances along probability threshold value). It comes with an illustration of the binary prediction (red shapes) performed for the corresponding T value, in the region of New Netim (Nigeria). Note that figures are computed for the whole *test set*, contrary to the sample images that aim to illustrate the effect of the T value variation on a relevant area. *Sources: Sentinel-2 (from the Copernicus program) & author calculations*.

We start by introducing the *precision* and *recall* statistics. They depict, respectively, our ability to i) correctly discriminate the mines—that is *precision*—and ii) to detect all of them—that is *recall* -, which are competing abilities though complementary. They are derived from the number of pixels correctly predicted as asm (true positives, TP, see Fig 2a), on the number of pixels that were mistakenly predicted as asm (false positives, FP), and the number of pixels that were mistakenly predicted as not asm (false negatives, FN).

$$\begin{array}{c} precision=\frac{TP}{\left(TP+FP\right)}\in \left[0:1\right]\;\;\;\;\;recall=\frac{TP}{\left(TP+FN\right)}\in \left[0:1\right] \end{array}$$

As their opposition is inherent to their conflicted purposes, we determine a relevant trade-off between those two statistics considering i) the upstream model’s objectives; ii) the positive class distribution; and iii) its potential ability to be distinguished from other classes of features. Consequently, we introduce the commonly used *F*(*β*)_*score*_, defined as the weighted harmonic mean between *precision* and *recall* [17]. Given that asm is a highly minority class, easily confused with many other features—such as any landscaping projects, partly due to the spatial resolution of the input satellite data -, we set the *β* parameter to 0.5, thus putting more emphasis on the predictions’ accuracy rather than their quantity. This ensures that the data provided has satisfying minimum levels of precision, reducing the false positives in priority (this indicator is also used to select the model’s final weights during the validation phase).

$$F{\left(\beta \right)}_{score}=\frac{(\beta^{2}+1)*precision*recall}{\left(\beta^{2}*precision+recall\right)}\in \left[0:1\right]\;\;,\;\;\beta >0$$

However, by ignoring the true negatives (TN) class in its computation, i.e. by focusing mainly on the positive class, the *F*(*β*)_*score*_ could present an incomplete view of the prediction reality [18]. As such, we suggest an alternative metric with the *Matthews Correlation Coefficient* (MCC). This standard metric, which evaluates the binary (two-classes) classification quality, has the advantage of often being described as a balanced measure as it can be used even if the classes are of very different sizes, i.e. in imbalanced classification problems such as asm prediction. See S5 Appendix in S1 File for details.

$$MCC_{score}=\frac{\left(TP*TN-FP*FN\right)}{\sqrt{\left(TP+FP)*(TP+FN)*(TN+FP)*(TN+FN\right)}}\in \left[-1:1\right]$$

### Performances along probability threshold value

A crucial aspect is the mapping of asm for different applications, varying from policy makers aiming to own a complete vision of all potential asm locations, to research use which requires highly accurate data. In this perspective, a major lever stands in the selection of the appropriate probability threshold (T, hereafter) to produce the final binary classification map from the model’s raw probability output.

As a matter of illustration, we distinguish three typical scenarios, illustrated on the region of New Netim, Nigeria (Fig 2b). In each scenario, we display the correctly predicted pixels on the matrix diagonal (TN and TP), which contains the vast majority of the *test set* surface, leading to an overall prediction accuracy larger than 99%, whereas on the anti-diagonal are counted the wrongly classified pixels (FN and FP). This matrix also highlights the class imbalance we have to deal with, assuming that the vast majority of the areas are not mines—and are classified as such correctly.

***T = 0.2*** (Fig 2b-i): In this scenario, we maximize the number of predicted locations where asm are likely to take place, while placing less emphasis on the precision of the information. This translates into an overview of at least 50% of the actual mines, but with a relatively low *precision* (around 60%). This scenario is particularly suitable when the information can be cross-checked locally, for instance by local policy makers or non-governmental organizations, as the data provided will contain false positives. As depicted on Fig 2b-i, mining areas are all detected as long with a mispredicted small part of a village.

***T = 0.4*** (Fig 2b-ii): This scenario stands as the middle ground situation between the maximum values for the *F*(*β* = 0.5)_*score*_ and the mcc. For instance, this scenario is perfectly suitable for the purpose of mapping overall asm activities when it is not possible to cross-check the information locally. Note that the small part of the village wrongly predicted in the previous scenario is not anymore.

***T = 0.6*** (Fig 2b-iii): Finally, this scenario corresponds to a maximum *precision* (≈90%) without a *recall* under 30%. For instance, this scenario is well suited to research projects focusing on the socioeconomic impacts induced by asm activities, for which the veracity of the asm locations stands as crucial, because it minimizes the false positives.

**Fig 3:**
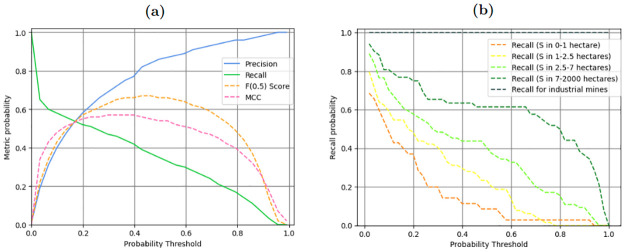
Main performance metrics computed on the *test set*. (a) Overall performance metrics (Precision, Recall, F(0.5) score and MCC) depending on the probability threshold value. (b) Recall metric along asm size classification depending on the probability threshold value.

Ultimately, Fig 3a) illustrates the evolution of the metrics with the T variation, hence demonstrating that choosing an appropriate T value goes along with relevantly selecting a specific set of metrics.

### Performances along ASM size classification

Another important feature of our model is its ability to detect asm independently from their ground surface range (in hectares). In our labelled dataset, the average asm size is around 13 hectares, while the standard deviation is around 70 hectares and 75% of the labelled asm cover less than 7 hectares (S3 Fig). This indicates that our model is trained on a wide range of asm sizes and not only large-scale (or industrial) mines. To investigate its ability to effectively detect this wide range of asm, we make use of the labelled dataset to create equally distributed mine-sized classes and compute the *recall* for each of them. We also add a last class to separately represent the industrial mining activities, since their size and features are distinct from asm. The resulting classes are: micro asm from 0.1 to 1 ha; small asm from 1 to 2.5 ha; medium asm from 2.5 to 7 ha; macro asm from 7 to 2000 ha; and separately the industrial mines.

Fig 3b) displays the results. Consistent with the previous performances, asm are more likely to be detected for low T values, and the *recall* decreases progressively as T increases, whatever the mine-sized class. Additionally, the larger the asm ground surface, the more easily it is identified by the algorithm, even though the large-scale mines are under-represented. Putting in perspective the results to pixels amounts to explain this phenomenon, micro asm only represent a few pixels, making the recognition essentially based on the colour bands, whereas macro asm also provide a typically recognizable mine shape and contain extra features—such as tailing ponds or large visible ditches via the variations in the pixels’ colours. Besides, as depicted on Fig 3b), the difficulty to detect the micro-asm (*recall* ≈ 12% for T = 0.4) supports our decision to favour the model’s predictions only for asm larger than 0.1 ha.

**Fig 4:**
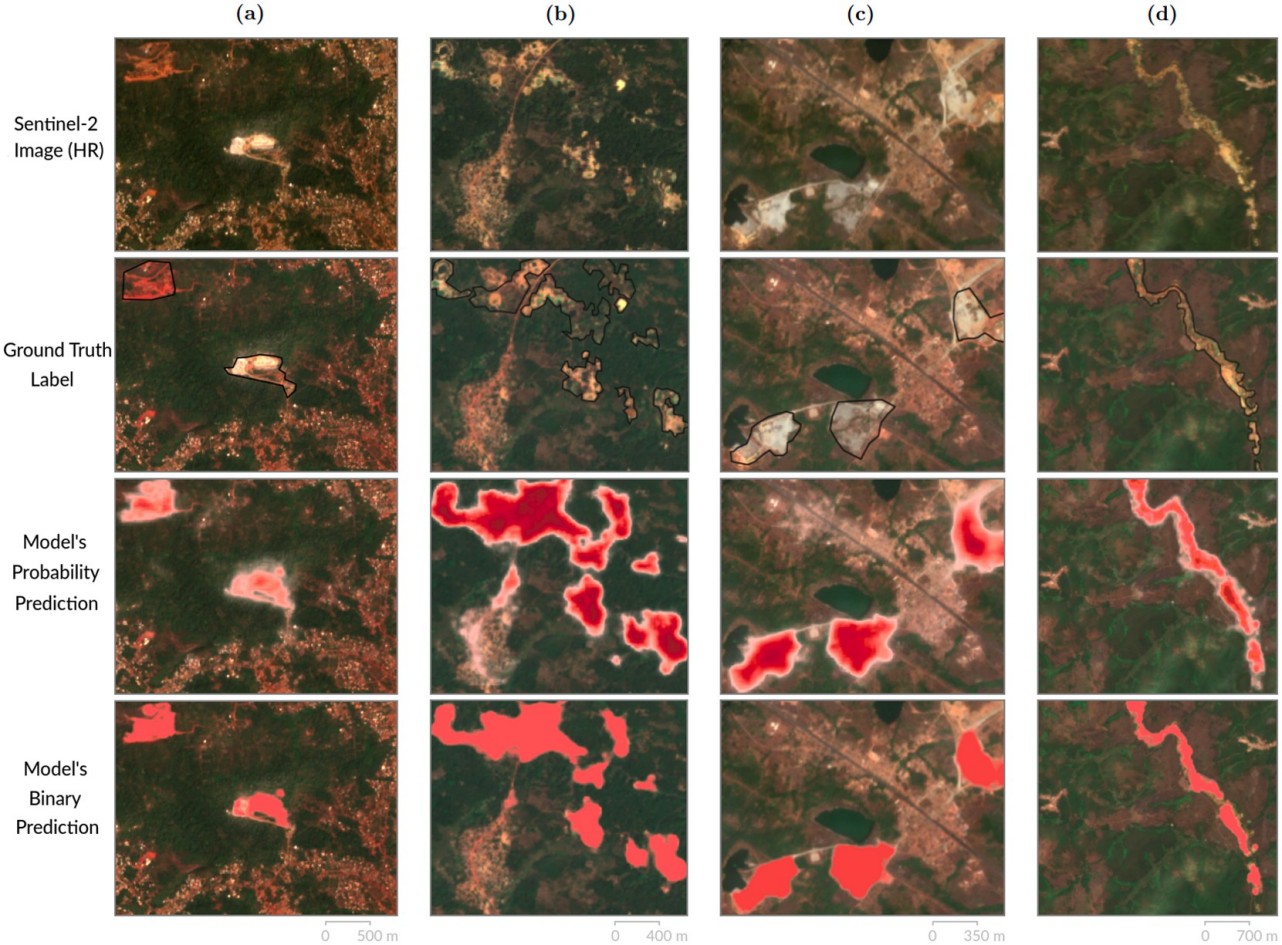
Timeline examples of various regions predicted by the model. Four examples of predictions performed by our model, from various parts of the studied region. Each vertical timeline chronologically presents i) a sample of Sentinel-2 (S2) model’s input image as reference, ii) the ground truth asm contouring label (in black) for this S2 sample, iii) the corresponding probability prediction performed by our model, and iv) the binary prediction obtained after applying a probability threshold T = 0.5. Sample a) comes from Yaoundé city in Cameroon (3°58’N: 11°30’E), sample b) comes from Kenema District in Sierra Leone (8°10’N: 10°56’W), sample c) comes from southern Nigeria (6°44’N: 5°09’E) and sample d) comes from Koinadugu District, Sierra Leone (9°09’N: 11°27’W). *Sources: Sentinel-2 (from the Copernicus program) & author calculations*.

Taken together, the results point out that our model performs well in detecting all kinds of asm—even with the smallest asm representing only a few pixels within images of 100 million pixels—with varying proportions depending on the selected scenario. This diversity is illustrated on Fig 4. Furthermore, selecting a specific scenario also implies querying specific mine-sized classes, which is another way to adapt the resulting data to the user’s particular objectives.

### Performances across climate and landscape variations

To envision extending the asm prediction to the whole African continent, the model’s robustness to landscape and climate diversity is the most crucial limiting factor. The two main biomes identified in our studied region (S1 Fig) respectively cover 54.2% (savannas, grasslands and shrub-lands) and 11.7% (moist broad-leaf forests) of the whole continent surface, and hence constitute a significant sample to determine the robustness of our model. Thus, we investigate the performances on each biome separately, by dividing the 27 boxes contained in our *test set* according to their biome affiliation. We observe coherent performances on the two major biomes: for *T* = 0.4, 85% *precision* while retrieving 46% of the asm on the moist broad-leaf forests biome (*F*(*β* = 0.5)_*score*_ = 0.73), and 60% *precision* while retrieving 28% of the asm on the savannas, grasslands and shrub-lands biome (*F*(*β* = 0.5)_*score*_ = 0.48). Note that the *recall* gap can be explained by i) the asm smaller average size and ii) by the lower contrasts between asm and ground vegetation in more arid regions such as savannas. Alternatively, the performances on the remaining biome—mangroves, less than 4% of the studied region and ≈ 0.1% of the whole Africa—are still too inaccurate, which can mainly be explained by its poor representation in our region. Still, the remarkable consistency of the results over the two major sub-Saharan eco-regions, which present very distinct and challenging landscape features (partly illustrated on Fig 4), indicates a comforting ability to generalize the procedure at very large scales.

## Discussion

### Comparison to literature

In this paper we use a convolutional neural network (cnn), which is widely adopted in the literature [10–13]. Alternatively, some authors use pixel-based classifiers to map asm activity in Ghana [19–21], Burkina Faso [22] and Brazil [23]. In opposition to cnn, pixel-based methods do not leverage the pixels’ context but only make use of their inherent properties limiting the detection of asm. Hence, their reported omission errors range from 8 to 40% for the mining class, indicating a large variability in the accuracy of this method [19–21]. In regards to the recent literature on mines detection using deep learning on the same spatial resolution (10m), our accuracy (> 99%) is similar but the performance of our model differs in some dimensions. First, covering 1.75 million km^2^ has the drawback to lower our segmentation metric in comparison to papers covering smaller regions, especially in the case of regions renowned for their rich content of asm, like Ghana with a region of 63,000 *km*^2^ covered [13]. Second, in contrast, our classification metrics are very close to recent coal mines classification [24], while detecting a larger array of mine’s types (Fig 4), which implies dealing with more heterogeneous data. Third, using very high resolution images (0.81m) to detect open-pit mines on a single region in China [25], they display a *F-Score* of 0.67 and a *precision* of 0.80. Our model’s performance is similar on the *precision* metric, but lower on the detection’s accuracy for mines smaller than 1ha. Besides the literature on mine detection, there is a growing literature that uses machine learning techniques and satellite imagery to measure various human or environmental outcomes with a very high accuracy [26]. For instance, with very high resolution images (1m) brick kilns are detected with a *precision* of 88% and with a very low probability to be missed [27], as well as trees in Sahel (0,5m) [28] that are detected up to a size of 3*m*^2^ with a very high *recall* of 95%. Last, our classifier achieves an Area Under Curve (AUC) of 0.86 on the *test set* (S4 Fig), which is similar and coherent with respect to the score of 0.84 obtained in [29] when detecting buildings’ destruction in conflict zones. To conclude, drawing a comparison to existing work highlights the necessity of making trade-offs between the area covered, the satellite resolution, the accuracy of the detection and the minimum size that can be detected.

### Results synthesis

By aggregating the results along the different dimensions, our model performs remarkably well compared to similar work that has been completed in this area, which reflects the relevant compromise made between cost, scale and performance criteria. Considering the case of a probability threshold of 0.4, the model predicts 1.75 million km^2^ for which 6,970 km^2^ are correctly reported as asm. First, we achieve a *precision* of 70.4% while reaching at the same time a *recall* of 42.2%. Second, detected asm activities range from 0.1 ha to around 2,000 ha, with for instance one-third of the small asm (1 ha < surface < 2.5 ha) retrieved, thus satisfying our primary ambition to detect the largest permissible range of asm. Third, we crucially achieve coherent performances on the two major biomes represented in this study. Furthermore, as the Mangroves area is not well predicted by the algorithm, if the user’s region of interest stands within the Tropical and sub-Tropical moist broad-leaf forests biome, predictions even get much greater: a *precision* of 85% while retrieving 46% of the asm and a satisfying MCC of 0.62. This last finding is particularly reassuring, suggesting our model should perform well on larger African regions or other continents. Additionally, we compare our predictions to the most exhaustive existing dataset on the large-scale mines locations (S&P Global—SNL Metals and Mining). We detect all of the 265 listed large-scale mines located within our region of interest. Moreover, we detect 700 clusters of asm, originally unrecorded, in a radius of 5km around the locations of the large-scale mines included in the S&P Global dataset.

## Conclusion

In this paper, we suggest a suitable strategy to detect asm activities ranging from 0.1 ha to around 2, 000 ha, on a region of 1.75 million km^2^ across 13 countries in Sub-Tropical West Africa. Our strategy succeeds in hitting acceptable compromises of performance, as achieving 70% precision while maintaining simultaneously 42% recall. We believe our approach, that has the virtue to be easily replicated, could be extended. For instance, it’s likely that the use of biome-targeted training, the integration of new spectral bands in input, making use of Sentinel-2’s temporal resolution, or using recent techniques such as transfer learning and weekly supervised learning, would upgrade the model’s performances or permit to broaden the scale covered (see S6 Appendix in S1 File for a detailed discussion). Our approach is also suitable to understand the history of mines and to detect new opening mines almost in real time—depending on the cloud coverage—thanks to Sentinel-2 high revisit frequency. Doing so, our approach echoes the call of Burke *et al*. (2021) [26] on the nine new areas of development expected in the remote sensing field to promote socio-economic and sustainable applications, by i) extending a specific methodology across large geographies while ii) enabling to measure changes over time. Eventually, this method offers a wide range of development applications, from making the monitoring of regions rich of asm an easier task, to research purpose by enlarging our knowledge of the consequences of asm activities.

## Supporting information

S1 Fig Tropical and Sub-Tropical Africa biomes repartition. Our region of interest (delimited by the black outline) covers three distinct biomes (also referred to as eco-regions), classified along the major habitat type as defined by World Wildlife Fund (WWF) [7]. The first biome—tropical and sub-tropical savannas, grasslands and shrub-lands (in orange)—covers 55.1% of the studied region. Second, the tropical and sub-tropical moist broad-leaf forests eco-region (in green) covers 41.1% of the studied region. Hence, the two main biomes together cover 96.2% of the region. Finally, the third biome identified is the Mangrove region (in blue). It only covers 3.8% of the studied region, while containing very specific features and landscapes.
(TIF)

S2 Fig Grid overview. The grid that is used to enumerate our labelled boxes has been created in such a way that the cells are overlapping, as depicted on this schema. One grid cell is highlighted (in green) along with a typical sub-cell to be extracted (in blue). The area in white between the blue boundary and the green boundary corresponds to the shared area between two neighbouring cells. As we use rotations of the original data to enlarge our dataset (augmentation process), we only extract a smaller square out of the maximal available zone to avoid no-data on the final image. Otherwise, the rotated square/cell would not cover the whole green area on the edges. Sharing these buffer zones with neighbouring cells hence ensures that they are still well represented in the final augmented dataset. A drawback of this approach is that the labelling data in those overlapping areas might have to be duplicated, as labelled mines are always associated to one grid cell.
(TIF)

S3 Fig Focus on mines’ sizes. (a) Cumulative Distribution Function (CDF) graph obtained on the labelled asm surfaces (in hectares) contained within our studied region, with a logarithmic representation for the sizes distribution. (b) Additional statistics computed on the same asm. As mentioned in the main part, the use of Sentinel-2 images—on which each pixel represents around 100 m^2^ at ground level—implies that only mines covering a minimum area of 1,000 m^2^ were reported in our reference datasets, which is then the minimum mine’s area retrievable in our case. At the contrary, the largest mining surface reported within the whole region covers an area of 2,000 hectares. This huge gap between upper and lower asm size boundaries points out the variety of mining areas we have to retrieve and therefore the need in quantifying if every kind of mining areas is well-retrieved, in other words the model’s robustness to mining shapes and sizes.
(TIF)

S4 Fig ROC curve. Receiver Operating Characteristic (ROC) curve with the Area Under the Curve (AUC) value obtained on our *test set*.
(TIF)

S5 Fig Studied region with Sentinel-2 tiles cover. The area of interest is covered by 222 overlapping Sentinel-2 tiles (green squares), each corresponding to a specific ID which are appearing on this visualization. *Sources: Copernicus (tiles grid)*.
(TIF)

S6 Fig Sentinel-2 pre-processing pipeline. Overview of the Sentinel-2 data pre-processing pipeline, which needs to be performed for each satellite image before being used to train the model: from the download part with Copernicus (left) via the bands manipulation (center) to get the operable normalized image (right). *Sources: Copernicus (Sentinel-2 images)*.
(TIF)

S7 Fig Feature maps visualization. (a) Activation in the bottleneck part of the model for each filter. (b) Ground truth associated with these feature maps. To interpret this, note that after the model training, the neurons are sensitive to mines on satellite images. Here, we can see that a lot of neurons are activated in the mining regions where others, probably specialized in detecting different types of mines, stay inactivated (black).
(TIF)

S8 Fig Detailed metrics obtained for the three scenarios. This figure presents the confusion matrices computed on the whole *test set* for three probability thresholds value: (a) T = 0.2, (b) T = 0.4 and (c) T = 0.6, as defined in the Results section. These correspond to a more detailed and traditional version of those presented in the paper (Fig 3), with corresponding surfaces (in ha) and accompanied with some metrics values (accuracy, precision, recall and F1-score).
(TIF)

S1 File (PDF)
