# Supplementary figures and images for: Mapping artisanal and small-scale mines at large scale from space with deep learning
Source: PLoS One. 2022 Sep 22;17(9):e0267963. doi: 10.1371/journal.pone.0267963 (PMC9498930; doi:10.1371/journal.pone.0267963)

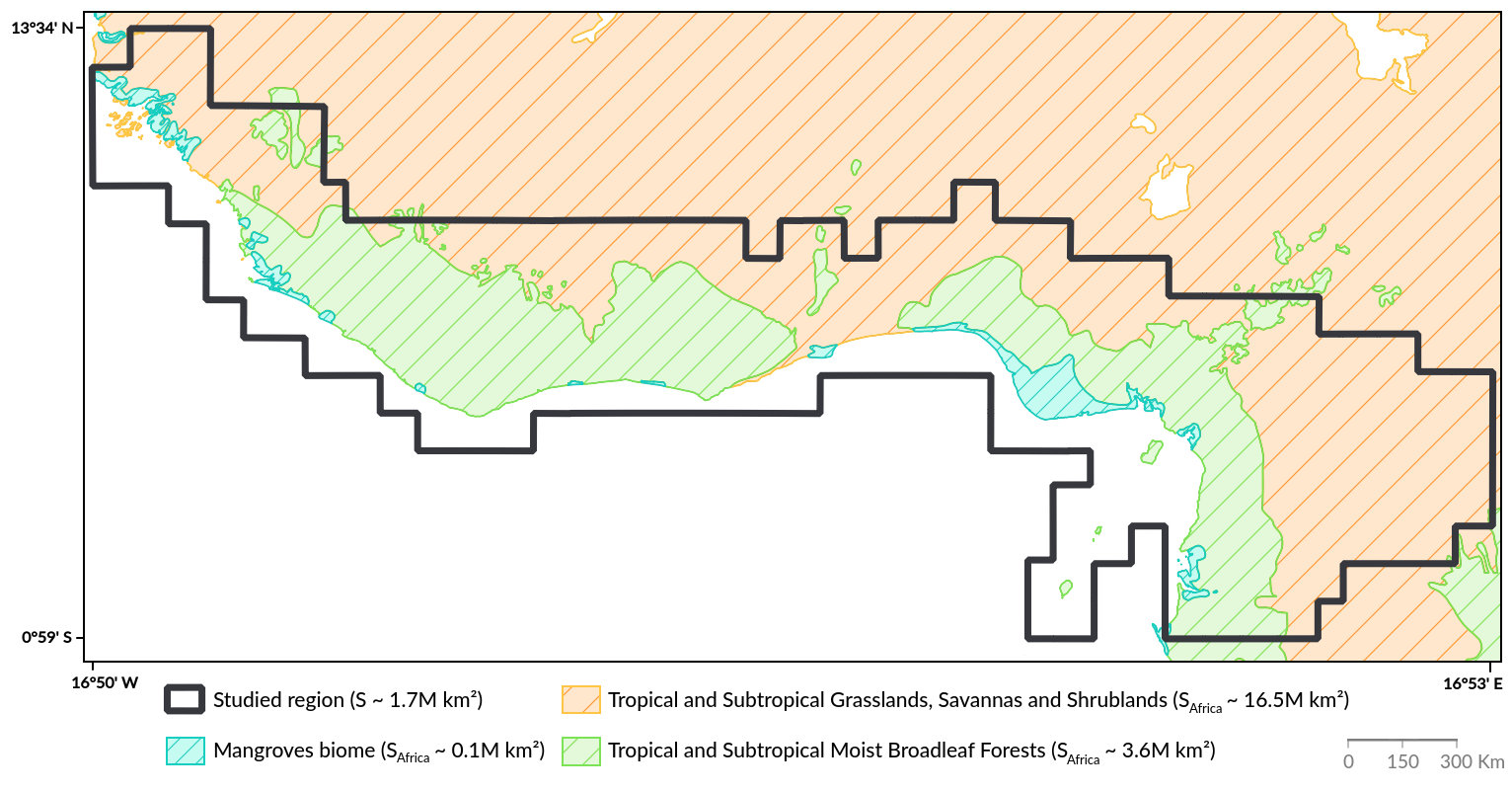

Supplement: S1 Fig — Our region of interest (delimited by the black outline) covers three distinct biomes (also referred to as eco-regions), classified along the major habitat type as defined by World Wildlife Fund (WWF) [7]. The first biome—tropical and sub-tropical savannas, grasslands and shrub-lands (in orange)—covers 55.1% of the studied region. Second, the tropical and sub-tropical moist broad-leaf forests eco-region (in green) covers 41.1% of the studied region. Hence, the two main biomes together cover 96.2% of the region. Finally, the third biome identified is the Mangrove region (in blue). It only covers 3.8% of the studied region, while containing very specific features and landscapes. (TIF) [file pone.0267963.s001.tif]

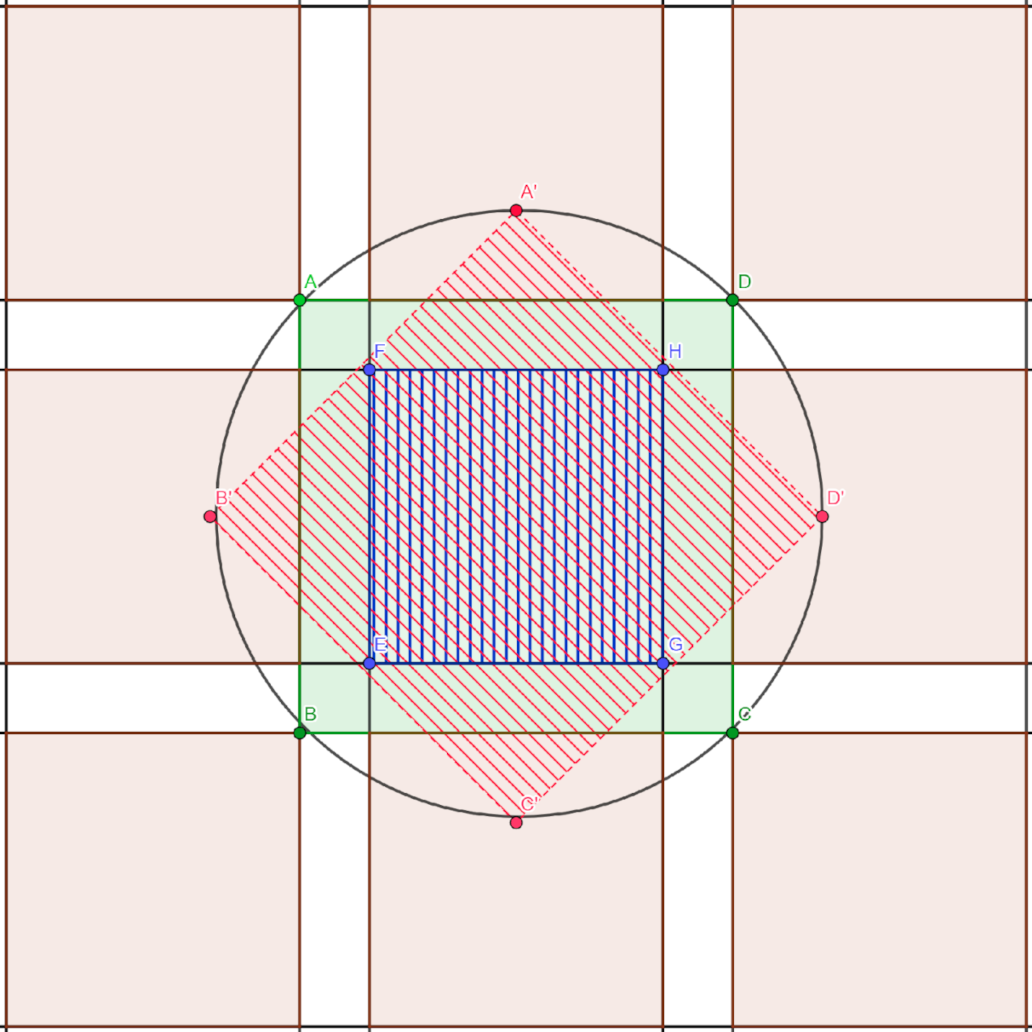

Supplement: S2 Fig — The grid that is used to enumerate our labelled boxes has been created in such a way that the cells are overlapping, as depicted on this schema. One grid cell is highlighted (in green) along with a typical sub-cell to be extracted (in blue). The area in white between the blue boundary and the green boundary corresponds to the shared area between two neighbouring cells. As we use rotations of the original data to enlarge our dataset (augmentation process), we only extract a smaller square out of the maximal available zone to avoid no-data on the final image. Otherwise, the rotated square/cell would not cover the whole green area on the edges. Sharing these buffer zones with neighbouring cells hence ensures that they are still well represented in the final augmented dataset. A drawback of this approach is that the labelling data in those overlapping areas might have to be duplicated, as labelled mines are always associated to one grid cell. (TIF) [file pone.0267963.s002.tif]

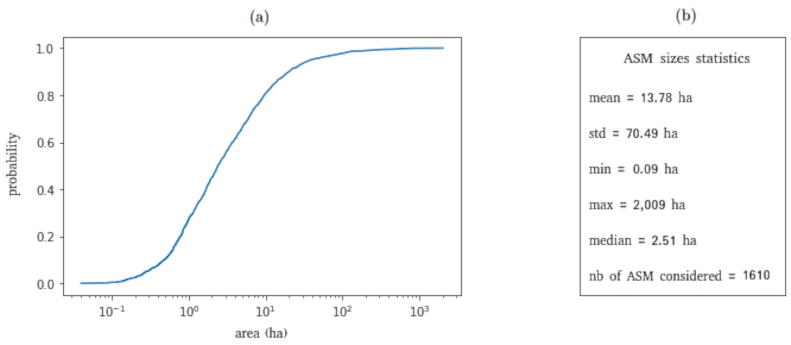

Supplement: S3 Fig — (a) Cumulative Distribution Function (CDF) graph obtained on the labelled asm surfaces (in hectares) contained within our studied region, with a logarithmic representation for the sizes distribution. (b) Additional statistics computed on the same asm. As mentioned in the main part, the use of Sentinel-2 images—on which each pixel represents around 100 m2 at ground level—implies that only mines covering a minimum area of 1,000 m2 were reported in our reference datasets, which is then the minimum mine’s area retrievable in our case. At the contrary, the largest mining surface reported within the whole region covers an area of 2,000 hectares. This huge gap between upper and lower asm size boundaries points out the variety of mining areas we have to retrieve and therefore the need in quantifying if every kind of mining areas is well-retrieved, in other words the model’s robustness to mining shapes and sizes. (TIF) [file pone.0267963.s003.tif]

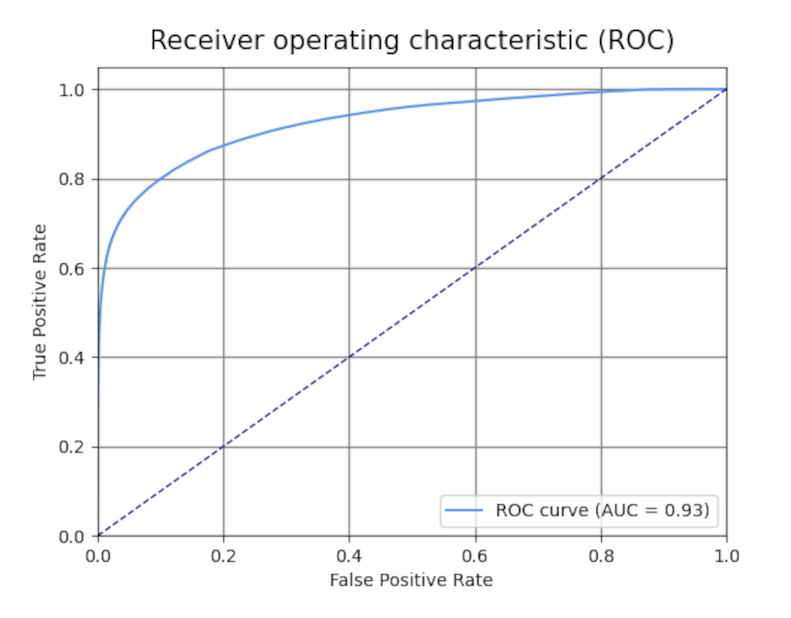

Supplement: S4 Fig — Receiver Operating Characteristic (ROC) curve with the Area Under the Curve (AUC) value obtained on our test set. (TIF) [file pone.0267963.s004.tif]

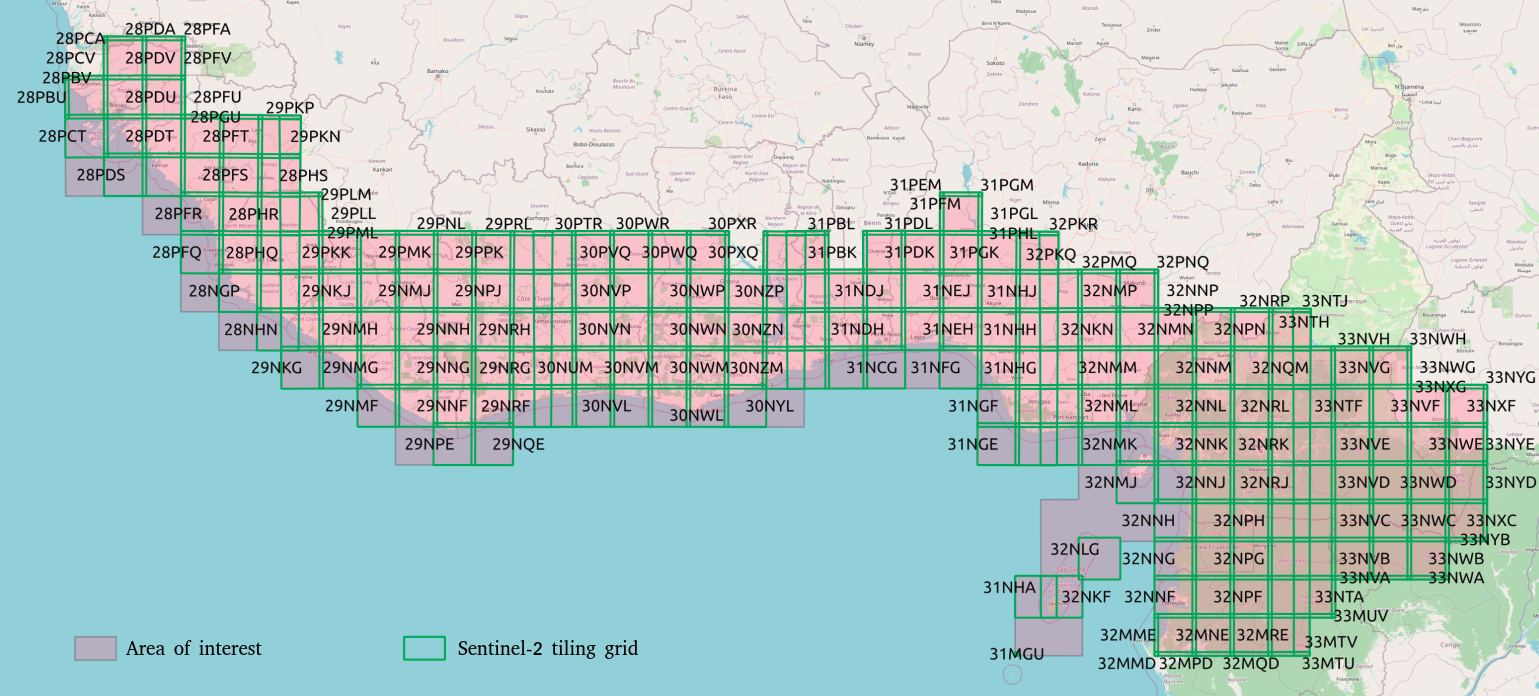

Supplement: S5 Fig — The area of interest is covered by 222 overlapping Sentinel-2 tiles (green squares), each corresponding to a specific ID which are appearing on this visualization. Sources: Copernicus (tiles grid). (TIF) [file pone.0267963.s005.tif]

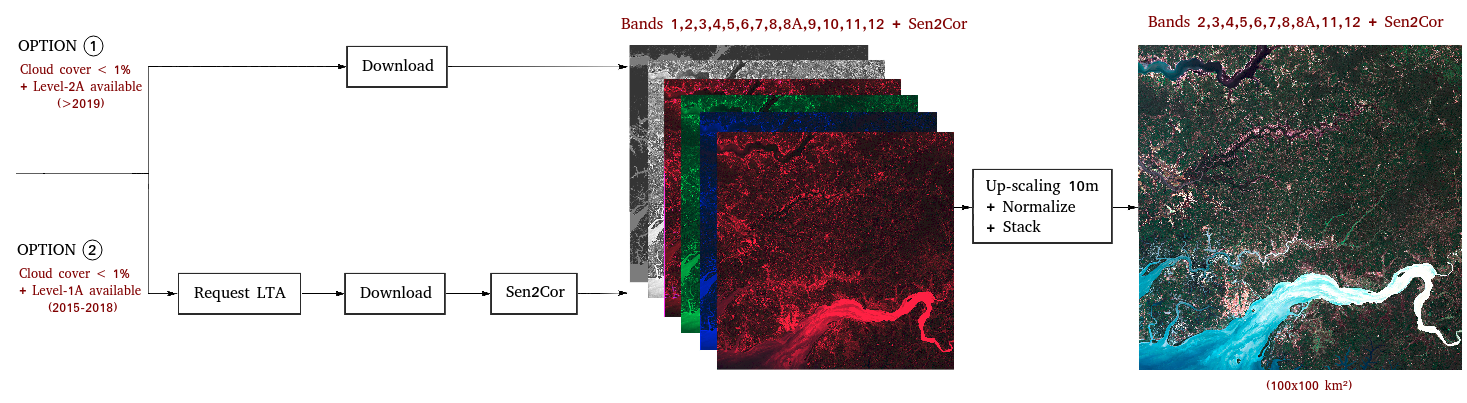

Supplement: S6 Fig — Overview of the Sentinel-2 data pre-processing pipeline, which needs to be performed for each satellite image before being used to train the model: from the download part with Copernicus (left) via the bands manipulation (center) to get the operable normalized image (right). Sources: Copernicus (Sentinel-2 images). (TIF) [file pone.0267963.s006.tif]

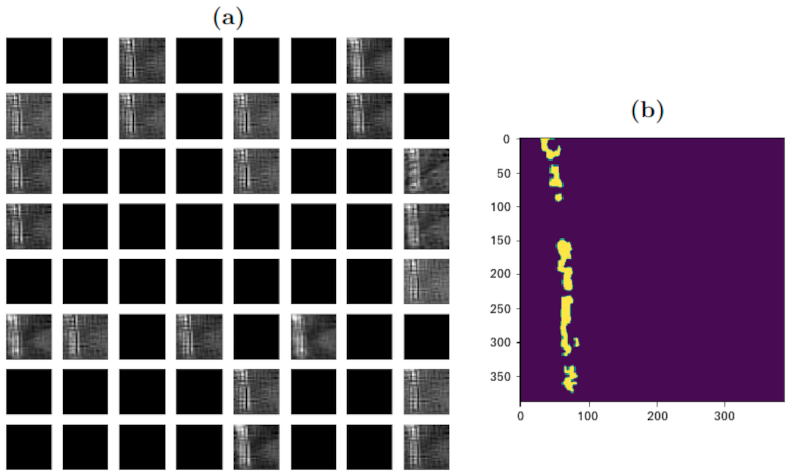

Supplement: S7 Fig — (a) Activation in the bottleneck part of the model for each filter. (b) Ground truth associated with these feature maps. To interpret this, note that after the model training, the neurons are sensitive to mines on satellite images. Here, we can see that a lot of neurons are activated in the mining regions where others, probably specialized in detecting different types of mines, stay inactivated (black). (TIF) [file pone.0267963.s007.tif]

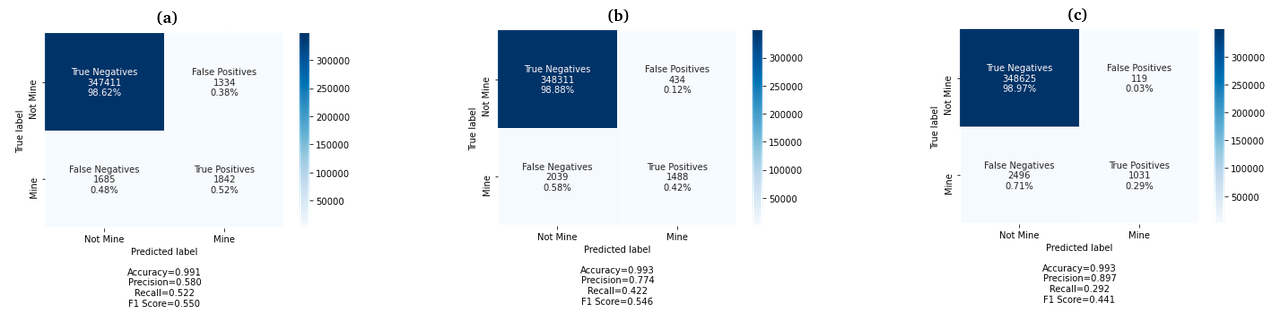

Supplement: S8 Fig — This figure presents the confusion matrices computed on the whole test set for three probability thresholds value: (a) T = 0.2, (b) T = 0.4 and (c) T = 0.6, as defined in the Results section. These correspond to a more detailed and traditional version of those presented in the paper (Fig 3), with corresponding surfaces (in ha) and accompanied with some metrics values (accuracy, precision, recall and F1-score). (TIF) [file pone.0267963.s008.tif]
