# Supplementary material for: Mapping artisanal and small-scale mines at large scale from space with deep learning
Source: PLoS One. 2022 Sep 22;17(9):e0267963. doi: 10.1371/journal.pone.0267963 (PMC9498930; doi:10.1371/journal.pone.0267963)
Supplement: S1 File — (PDF) [file pone.0267963.s009.pdf]

S1 Appendix. Motivation for the use of Sentinel-2. We make use of Sentinel-2 satellite images provided by EU's Copernicus program operated by the European Space Agency [1], with each pixel's resolution of 10m×10m. The satellite's instruments is made of a set of 13 spectral bands spanning from the visible light to the short-wave infrared (see S1 Table), hence constituting a reference service for land monitoring, emergency response and security services. Alternative sensors offer lower resolutions (like 3 meters resolution with PlanetScope) but the cost to cover a large-scaled region would be tremendous. Moreover, Sentinel-2 satellite images follow an open data policy by allowing all users to freely access high resolution data from 2015 to nowadays, with an attractive revisit period of 5 days in most parts of the world (including Africa), and the mission's operation is planned until 2025 (with a potential extension to 2030), making the use of Sentinel-2 a reliable and sustainable solution for several years.

S2 Appendix. Sentinel-2 data. The project's scalability represents a major technical challenge, as the area covered represents 222 different Sentinel-2 tiles - one tile matches 100km×100km at ground level - (S5 Fig): 100 tiles are required to create the labelled dataset and the remaining tiles being a mixture of uncompleted / too cloudy tiles, or tiles used to test other methods, such as enhancing the training set with temporal data. Thanks to the Copernicus program policy, Sentinel-2 images are freely available [1], but still with some limitations:

- According to their product retention policy, Sentinel-2 images products are kept online according to a sliding window period of around one month. After this period, the image is archived and needs to be explicitly queried before being able to download it. Building a system to keep automatically track of Long Term Archive for all images before 2020 on Copernicus bib29 [1] is required.
- Sentinel L2A level images were rarely available. We only stored L1C level images and processed them ourselves automatically with Sen2cor on our infrastructure to transform them into L2A (cf S3 Appendix).

To give an insight of the storage capacity needed, once processed, stacked and normalized, each Sentinel-2 tile weights approximately 6 Go. Additionally, in case of failure during the processing pipeline, it is recommended that each processing state of the tile is stored separately : downloaded archive ($\sim$ 800 Mo), extracted Level-1C product ($\sim$ 800 Mo), L2A level product ($\sim$ 1 Go) and finally its stacked ($\sim$ 3.1 Go) and normalized ($\sim$ 6.3 Go) versions. In our case, these tiles are stored in Hard Disk Drives (HDD) of 12TB and 2×8Tb that are duplicated for backup.

S3 Appendix. Images pre-processing overview. In order to use the satellite images as input for our model, we apply the following four-steps pre-processing methodology on the downloaded tiles ($100 \times 100\ km^2$): first, we atmospherically and geometrically correct the Sentinel-2 images thanks to the *Sen2Cor* program. *Sen2Cor* also creates a scene classification map (SCM) that maps each pixel into various categories, like *cloud* or *water* (S2 Table). Second, bands with a resolution of $20m \times 20m$ are upscaled to the joint resolution of $10m \times 10m$, and spectral bands with a resolution of $60m \times 60m$ are discarded (S2 Fig). Third, we transform the mentioned classification bands into 12 one-hot encoded bands (binary value per pixel and per band, each band respectively indicating the presence of clouds, water or defective data, cf S2 Table). Fourth, we normalize the image by mapping the histogram's 2nd and the 98th percentiles to $[-1, 1]$ for each spectral band (each pixel considered as *saturated or defective* or *cloud high and medium probability* are excluded from the percentile calculation). The interval for cutting the histogram was chosen upon visual criteria. As it greatly helps the human eye to detect features on the satellite images, we expect it to improve the parameter learning for the model as well. A visualization of the processing pipeline is available on S6 Fig. To train our model, we apply additional steps for data augmentation: we extract the data from our training set regions (14.5km × 14.5km, each) with different rotations (15, 30 and 45 degrees) and divide them into overlapping patches of 572×572 pixels (overlapping as model's input and output sizes are different). Ultimately, during the training, various random transformations are applied to the patches with a given likelihood - flipping, brightness and contrast modifications, transpositions and 90 degrees random rotations - thanks to the Albumentation image augmentation library [2].

S4 Appendix. Detailed overview of the U-Net model. We make use of a modified version of the deep learning model U-Net [3]. One should notice that this model's input size (572×572 pixels) is larger than the model's output size (388×388 pixels) as the convolutions are unpadded, to avoid zero padding and to rather use all of the context's richness from the images, which was pertinent in our case. In practice, we have tested various model versions before to set its final shape. Starting with a "vanilla" U-net, then implementing slightly different variations by adding and/or removing layers and/or features, adding residual blocks [4], and finally adding batch normalization and dropout layers. It appears that the most efficient version of the model was the original structure, only enhanced by the two latter adds. Other versions either gave similar performances while generating higher complexity, either resulted in gradually poorer performances when departing too far from the initial model's structure. Also, we have tested using the original U-net without any dropout nor batch normalization layers. At the end, all ended with higher metrics on our training data but much worse metrics on our validation data, which was a clear sign of over-fitting. Hence, our model differs from the standard U-Net model by having additional batch normalization layers [5] and dropout layers [6], with a dropout probability considered worth 0.3, to reduce its initial tendency to over-fit on the training data. Intrinsically, this dropout layer randomly sets some of the network's neurons to 0 during training, so that iteratively the model is forced to activate all the neurons. The effect is that the network becomes less sensitive to specific neurons, resulting in a better generalization, which was mandatory for our purposes. For training, we used a focal loss function [7] (with parameters $\gamma = 2$, $\alpha = 0.25$) combined with the $Adam$ optimizer (learning rate $= 1.0 \times 10^{-3}$) [8]. Even if the latter stands as a widely used and cross-validated tool in deep learning models calibration [9, 10], other losses were tested at first, like the usual binary cross-entropy, the tversky loss [11] and the focal tversky loss [12]. Finally, the focal loss is the best performing loss function, as it was designed for imbalanced dataset problems just like ours. Additionally, we made use of the bib37 [13] python library which optimizes neural networks hyperparameters to get the most accurate results. With this library, the hyperparameters are still chosen by hand, however it allows to pick an initial range of values to be tested for each of them. The ones leading to the best results are saved, by testing them iteratively on a few epochs. The optimized parameters ranges are: the learning rate $[1.0 \times 10^{-4} : 1.0 \times 10^{-3}]$, the number of filters used on each convolution layer $\{16; 32; 64\}$, the dropout rate $\{0.1; 0.3; 0.5\}$ and the size of the kernel in convolutions $\{3; 5; 7\}$.

Concerning the U-net model's structure more specifically, it consists of an U-shaped encoder-decoder structure, with the contraction in the beginning, the bottleneck in the middle and the expansion part in the end. The contraction part is made of multiple convolution and pooling layers. These blocks down-sample the image while extracting the sharpest features at the same time. The number of features is doubled after each down-sampling so that the model can learn more effectively the complex structures of the image. The bottleneck part is the bottom part of the "U" which is needed to link the contracting and expanding parts. This part corresponds to the final compression of the input data, meaning this view contains only the primary useful information needed to be able to reconstruct the segmentation map based on the input. S7 Fig displays an example of what was obtained at the bottleneck, i.e. what are called the feature maps. Some of them have a strong value on the left side, with a shape similar to what the ground truth looks like, which means the ASM have been successfully recognized as so. The expansion part is quite similar to the contraction but the down-sampling becomes an up-sampling of the image. However, the input of each expansion block is concatenated to the corresponding contracting block in order to reconstruct the image with context information from the higher resolution layers. Eventually, the model is applied a 1×1 convolution with a binary cross-entropy activation which gives to each pixel of the output image a probability value between 0 and 1 of being a mine.

S5 Appendix. Overall Metrics. In this paper, are referred as True Positives (TP) the pixels correctly predicted as mining areas - i.e. pixels that were defined as a mining area in ground truth -, as True Negatives (TN) the pixels correctly predicted as not mining areas, and as False Positives (FP) and False Negatives (FN) the pixels that were mistakenly predicted as mines and not mines respectively. In Machine Learning analysis, these are the core statistical results - from which the majority of the other metrics derives from - traditionally presented within a Confusion Matrix (CM) as illustrated on S8 Fig. From the CM can be computed a wild number of metrics.

For the $F(\beta)_{score}$, the β parameter ($\beta > 0$) enables us to state which of *precision* or *recall* is the priority performance, setting β to 1 results in looking for perfect balance between *precision* and *recall*, whereas $\beta > 1$ results in focusing on the *recall*, and on the contrary $\beta < 1$ results in giving priority to the *precision*. Note that the $F(\beta)_{score}$ ranges between 0 and 1, the higher the value, the higher the model's performance. Concerning the MCC, it behaves like a traditional correlation coefficient: 0 meaning that the model's performance is no better than with a random model; 1 if the model perfectly predicts all the pixels; and -1 pointing out a total disagreement between the prediction and the ground truth.

Another interesting computed metric is the ROC Curve, which gives us the trade-off between the True Positive Rate (TPR) and the False Positive Rate (FPR) for various probability thresholds. It also provides a measure comparable to other models via the computation of the area under this curve (AUC) score (S4 Fig).

S6 Appendix. Promising improvement leads. Several directions for improving the performances and/or extending the area covered may be considered. The first path would be to conduct some biome-targeted specific training. From our experience, we expect that using images having less diverse backgrounds would greatly strengthen the model's ability to distinguish the mines from the other landscapes. It requires having enough example data into each biome separately, and hence processing larger areas than our current studied region. The second further improvement stands in the use of temporal coverage. We may feed the model with images from the same places but at a slightly different time (for instance, into a 1 month window around the original date), while keeping the same ASM labelling. It would drastically enlarge the training data easily, but at the cost of assuming that the labelled ASM shapes do not change over time, hence resulting in a decrease of our labelled data accuracy. Third, we think promising to use weakly supervised training technology [14] to fine-tune the model's performances, by making use of the worst recurring predictions spotted. One can imagine to implement this several times, by incrementally recreate a new training set containing the latest worst errors observed from each latest prediction, with for instance varying proportions of ASM, or with varying proportions of each landscape / feature. Note that contrarily to the evolving training sets, the validation and test datasets must remain unchanged in order to assess trustworthy performances analyses. Fourth, by adding or improving pre- and post-processing filters (e.g. a cloud or river mask), which would assume a trade-off between removing all possible false positive occurrences while at the same time removing

some accurate predictions. Fifth, integrating alternative physical information in the input bands, such as radar or telemetry data, would certainly help in spotting the most steep mines, at the cost of adapting this kind of data to the current Sentinel-2 format. Last, we envision extending to larger regions by making use of transfer learning from the most ”easy” (i.e. vegetated) biomes to the most “difficult” (i.e. arid) ones, hence overcoming the challenge of discriminating the mines within plain-colored landscapes.

S1 Table. Spectral bands of Sentinel-2 images. Characteristics of the spectral bands perceived by Sentinel-2 sensors. The bands shaded in light gray are excluded from our model's inputs as the resolution is too low and as they are mostly measuring atmospheric properties.

Spectral band	Central wavelength $[nm]$	Spatial resolution $[m]$
band 1 (coastal aerosol)	442	60
band 2 (blue)	492	10
band 3 (green)	559	10
band 4 (red)	665	10
band 5 (Vegetation red edge)	704	20
band 6 (Vegetation red edge)	740	20
band 7 (Vegetation red edge)	781	20
band 8 (NIR)	833	10
band 8A (Narrow NIR)	864	20
band 9 (Water vapour)	944	60
band 10 (SWIR - Cirrus)	1,375	60
band 11 (SWIR)	1,612	20
band 12 (SWIR)	2,194	20

S2 Table. Sen2Cor classification. Overview of the classes produced by the *Sen2Cor* application and their corresponding values.

Value	Classification
0	NO_DATA
1	SATURATED_OR_DEFECTIVE
2	DARK_AREA_PIXELS
3	CLOUD_SHADOWS
4	VEGETATION
5	NOT_VEGETATED
6	WATER
7	UNCLASSIFIED
8	CLOUD_MEDIUM_PROBABILITY
9	CLOUD_HIGH_PROBABILITY
10	THIN_CIRRUS
11	SNOW

References

1. url=https://bib29.copernicus.eu/
2. Buslaev, Alexander and Iglovikov, Vladimir I. and Khvedchenya, Eugene and Parinov, Alex and Druzhinin, Mikhail and Kalinin, Alexandr A. Albumentations: Fast and Flexible Image Augmentations Information 2020
3. Ronneberger, Olaf and Fischer, Philipp and Brox, Thomas U-Net: Convolutional Networks for Biomedical Image Segmentation 2015
4. Kaiming He and Xiangyu Zhang and Shaoqing Ren and Jian Sun Deep Residual Learning for Image Recognition 2015
5. Sergey Ioffe and Christian Szegedy Batch Normalization: Accelerating Deep Network Training by Reducing Internal Covariate Shift 2015
6. Srivastava, Nitish and Hinton, Geoffrey and Krizhevsky, Alex and Sutskever, Ilya and Salakhutdinov, Ruslan Dropout: A Simple Way to Prevent Neural Networks from Overfitting Journal of Machine Learning Research 2014
7. Tsung-Yi Lin and Priya Goyal and Ross Girshick and Kaiming He and Piotr Dollár Focal Loss for Dense Object Detection 2018
8. Diederik P. Kingma and Jimmy Ba Adam: A Method for Stochastic Optimization 2017
9. Libo Wang and Rui Li and Chenxi Duan and Ce Zhang and Xiaoliang Meng and Shenghui Fang A Novel Transformer based Semantic Segmentation Scheme for Fine-Resolution Remote Sensing Images 2021
10. Adam Paszke and Abhishek Chaurasia and Sangpil Kim and Eugenio Culurciello ENet: A Deep Neural Network Architecture for Real-Time Semantic Segmentation 2016
11. Seyed Sadegh Mohseni Salehi and Deniz Erdogmus and Ali Gholipour Tversky loss function for image segmentation using 3D fully convolutional deep networks 2017
12. Nabila Abraham and Naimul Mefraz Khan A Novel Focal Tversky loss function with improved Attention U-Net for lesion segmentation 2018
13. url=http://bib37.github.io/bib37/
14. Ienco, Dino and Gaetano, Raffaele and Gbodjo, Yawogan Jean Eudes and Interdonato, Roberto Weakly Supervised learning for land cover mapping of satellite image time series via attention-based CNN IEEE Access 2020
